# Synergistic antibacterial effects of *Ulva lactuca* methanolic extract alone and in combination with different antibiotics on multidrug-resistant *Klebsiella pneumoniae* isolate

**DOI:** 10.1186/s12866-023-02854-5

**Published:** 2023-04-18

**Authors:** Abeer I.M. EL-Sayed, Mostafa M. El-Sheekh, Mofida E.M. Makhlof

**Affiliations:** 1 Botany and Microbiology Department, Faculty of Science, Damanhour University, Damanhour, Egypt; 2 Botany Department, Faculty of Science, Tanta University, Tanta, 31527 Egypt

**Keywords:** *Ulva lactuca*, Antibacterial, MIC, Synergism, Antibiotics, *Klebsiella pneumoniae*

## Abstract

Various antibiotics are available, including gentamicin, chloramphenicol, ampicillin, amoxicillin, and streptomycin, but they have some restrictions. Many microorganisms are resistant to these medications. A new antimicrobial source must be found or developed to solve this issue. Inhere, extract from seaweeds *Ulva lactuca* was investigated for its antibacterial activity using a well diffusion assay against *Klebsiella pneumoniae*, and a promising inhibition zone diameter was recorded to be 14.04 mm. The biochemical structure of the antibacterial compound was determined via GC-MS and FTIR analysis. Also, a micro-dilution assay was used to calculate the minimum concentration that makes inhibition (MIC) to be 1.25 mg/ml from *U.* extract reliable to prevent the visibility of any bacterial growth, this was followed by examining the antibacterial effect of *U. Lactuca* methanolic extract alone and the synergetic effect of *U. Lactuca* methanolic extract in combination with two different antibiotics (gentamicin and chloramphenicol). This was assayed by the agar well diffusion method to achieve promising and strong inhibiting power against *K. pneumoniae.* It was deduced that the maximum synergism could be achieved by adding 2.5 mg/ml of *Ulva* methanolic extract to gentamicin (4 µg/ml), and the results were illustrated obviously via transmission electron microscope in which severe morphological deteriorations were experienced by the treated cells. From this study, we can conclude that *U. lactucae* extract has the power to aid antibiotics in reducing the growth of pathogenic *K. pneumoniae*.

## Introduction

One of the most well-known sources of bioactive substances is marine algae. Many seaweeds release bioactive substances that prevent the development of gram-positive and gram-negative bacteria [1–4]. Using antibacterial chemicals from natural products, such as extracts of marine macroalgae, has received attention. Recent advancements have shown that compounds extracted from seaweeds are more effective antimicrobial agents that prevent infectious diseases and microbial contaminations [5–[6]–[7]–[8]–9].

*Ulva* is a form of edible algae present on the oceanic shores of all continents [10]. Their fascinating chemical makeup makes commercial exploitation appealing for creating functional or healthy food [11]. *S. aureus* and *P. aeruginosa* are frequently seen in human diseases and are sensitive to the seaweed *U. fasciata’s* antibacterial capabilities [12]. Although it is unclear how well *U. lactuca* extracts work as an antibacterial product against *Staph. aureus* clinical strains [13]. Secondary metabolites in *U. lactuca* include alkaloids, triterpenoids, steroids, saponins, phenolic compounds, and flavonoids [14]. Because of their antibacterial, anti-inflammatory, antioxidant, and anticoagulant properties, these active substances may hasten wound healing in nosocomial wound infections [15, 16]. The mechanism of *U. lactuca* a promising antibacterial as well as an anti-inflammatory agent in wound healing is poorly understood [17].

*Klebsiella pneumoniae* used to be a strong cause of lung inflammation disease and meningitis. Nowadays, a hazardous disease called pyogenic liver abscess “caused by *K. pneumoniae*” has received great attention; Immunocompromised patients, infants, and the elderly are at a high risk of infection by these diseases. This bacterium has emerged as a significant and potentially dangerous pathogen in nosocomial and community-acquired infections. The pathogenicity factor that caused this bacterium’s unforeseen spread is a thick capsule that encases the entire cell [18]. Several multi-resistant *K. penumoniae* strains could describe a protective layer to the antibiotic treatment protocol, which included wide and extended-spectrum antibiotics, causing the severe spread of *K. pneumoniae* infections worldwide. As a result of this challenging global threat, new sources of valuable and available naturally produced compounds should be discovered to remedy such pathogens.

Multi-drug resistant (MDR) strains have emerged due to the increased use of antimicrobial drugs to treat bacterial illnesses. This information on the genetics and mechanisms of bacterial resistance could be used to develop measures to mitigate the effects of antimicrobial resistance [19]. A unique and promising idea, drug synergism between existing antibiotics and bioactive extracts may have positive (synergistic or additive interaction) or negative (deleterious) effects (antagonistic or toxic outcome) [20]. Therefore, the objective of the current study is to evaluate antimicrobial, minimum inhibitory concentrations, and the synergetic effect of *Ulva lactuca* methanolic extract alone and in addition to several antibiotics to inhibit an opportunistic pathogen, *Klebsiella pneumoniae* RCMB 003 (1) ATCC 13,883 with a full illustration of morphological cell changes via transmission electron microscope.

It is worth noting that for the first time, *U. lactuca* methanolic extract is tested against multidrug-resistant *Klebsiella pneumoniae* RCMB 003 (1) ATCC 13,883 in combination with different antibiotics.

## Experimental

### Alga Collection and processing

*Ulva lactuca*, from the family Ulvaceae, was used in this study, identification of algal species was made as presented in the literature and following Aleem [21]; Aleem [22]; Lipkin and Silva [23] and confirmed using Algae Base website M.D. Guiry in Guiry [24]. Handy picking of the tested Sample was done from the rocky areas of Abu Quir Bay, Alexandria, Egypt. Alga was washed after collection several times with seawater to get rid of adhering debris, associated biota, and sand, then under tap water to wash salts. In the shady area, the algal samples were dried by air, then dried in an oven (Memmert, Germany) at sixty ^o^C for three hours. The dried samples were ground into fine particles by a coffee grinder (Brown mill) and then stored for further experiments in plastic bags at room temperature.

### Preparation of ***U. lactuca*** methanolic extract

A dried alga sample was soaked in methanol at a ratio of 1:10 for three days, then filtrated, and the process was repeated three times until full extraction. After collecting the filtrates, the solvent was evaporated using a rotary evaporator set to 50 degrees Celsius. (Büchi, Switzerland). Weighted crude extracts were suspended in 50 mg/ml of dimethyl sulfoxide (DEMSO) and then chilled [25].

### Gas chromatography-mass spectrometry (GC–MS)

The composition of *U. lactuca* methanolic extract was determined using GC-MS (Thermo Scientific TRACE 1310 Gas Chromatograph attached with ISQ LT single quadrupole Mass Spectrometer). The *Ulva* extract was desorbed in a GC injector at 200 ºC and detector temperature at 300 ºC for 5 min in splitless mode and chromatographic. Column, DB5-MS, 30 m; 0.25 mm ID (J&W Scientific). The GC oven temperature was programmed from 40  ºC (3 min) − 280 ºC (5 min) at 5 ºC/min. -290 ºC (1 min) at 7.5 ºC/min. Helium carrier gas was employed, at a constant 1 ml/min flow rate of.

### FTIR

FTIR was done to distinguish the active compounds in the *Ulva lactuca* methanolic extract. This method involves obtaining the sample’s peaks using infrared radiation (IR), that passed from 103 − 100 cm^− 1^ times through the algal extract [26]. Some IR rays are transmitted while others pass through the sample. The sample of algal extract used the IR radiation it had absorbed and turned it into energy [27]. The FTIR results show the peaks of transmittance and absorbance. The range of the generated spectrum can be between 4000 and 500 cm^− 1^ [28].

### Assays on bacteria

#### Bacterial strain

Referenced pathogenic bacterial strain *Klebsiella pneumoniae* RCMB 003 (1) ATCC 13,883 was used in this study (RCMB) stands for Regional Center for Mycology and Biotechnology. First, the diseases this bacterium causes led to the choice of this pathogen. Additionally, this bacterium has become resistant to the available antibiotics.

#### Antibacterial assay of ***Ulva lactuca*** methanolic extract

The pathogenic bacterium was utilized to test the algae methanolic extract’s antibacterial activity using the agar well diffusion method [29]. Preparing the inoculums for the antibacterial experiment need pure colonies of *Klebsiella pneumoniae* RCMB 003 (1) ATCC 13,883 that were grown in nutritional broth media for 24 h. The Mueller Hinton Agar (MHA) medium was made, autoclaved for sterilization, and then distributed evenly into Petri plates. To create a homogeneous layer of the bacterial suspension, 100µL of the pathogenic microbe culture broth (CFU 10^6^ cells/mL) was delicately dispersed into various sterilized Petri plates using a sterile glass spreader. Each plate was shaped into a well with a diameter of 6 mm using a sterile cork borer before being loaded with the standard antibiotic gentamicin (4 µg/ml) (as reference) and *Ulva lactuca* methanolic extract. Gentamicin solution was created using sterile distilled water and gentamicin at 4µ g/mL concentrations. Petri dishes were closed and kept at 37 °C in an incubator. Clear inhibition zones were visible surrounding the wells in the Petri plates after 24 h, and their diameter (in mm) was observed. Data were provided as mean ± standard deviation (SD), and the experiment was carried out in triplicate.

#### MIC determination

*Ulva lactuca* methanolic extract MIC with standard antibiotic gentamicin (4 µg/ml) was examined using a micro-dilution assay [30]. Different concentrations of the algal extract and antibiotic were prepared using 5% DMSO. Concentrations of (1.25, 2.5, and 5 mg/mL) from *Ulva* extract with antibiotic (4 µg/ml) were homogenized in Mueller Hinton broth (MHB) tubes inoculated with 100 mL of pathogenic bacterium (1.5 × 10^8^ CFU/mL). The controls were tubes of MHB inoculated with examined bacterium and incubated for 24 h at 37 ℃. The MIC values were calculated as the lowest dose of antibacterial substances that stopped *Klebsiella pneumoniae* RCMB 003 (1) ATCC 13,883 from growing visibly [31].

#### Synergetic effect between ***Ulva lactuca*** extract and antibiotics against ***Klebsiella pneumoniae*** RCMB 003 (1) ATCC 13,883

The method of agar well diffusion (AWDA) was done to check the synergistic effect of *Ulva lactuca* methanolic extract (1.25 mg/ml and 2.5 mg/ml) with two different antibiotics (Gentamicin 4 µg/ml and Chloramphenicol 500 µg/ml) in controlling the growth of *Klebsiella pneumoniae* RCMB 003 (1) ATCC 13,883. Briefly, 100µ l of the activated culture of *Klebsiella pneumoniae* RCMB 003 (1) ATCC 13,883 were used to inoculate Mueller-Hinton agar plates, and five wells were made in each plate; one well for 500 µg chloramphenicol + 1.25 mg/ml *Ulva* extract, another well for 500 µg chloramphenicol + 2.5 mg/ml *Ulva* extract, a new well for 4 µg gentamicin + 1.25 mg/ml *Ulva* extract, fourth well for 4 µg gentamicin + 2.5 mg/ml *Ulva* extract and the last well for 1.25 mg/ml algal extract as control and put in an incubator at 37 °C for a day. Then, the zone of inhibition widths (ZOI) was determined. The following equation was used to determine the synergistic effect (SE):

1 $$SE = \frac{{B - A}}{A} \times 100$$

Where A is antibiotic ZOI and B is antibiotic + *Ulva lactuca* methanolic extract ZOI [32].

#### Transmission electron microscope

Electron microscopy was done to illustrate changes made in cells of pathogenic *Klebsiella pneumoniae* RCMB 003 (1) ATCC 13,883 before and after treatment by (highest MIC result); *Ulva lactuca* methanolic extract (2.5 mg/ml) in combination with antibiotic gentamicin (4 µg/ml). For TEM preparation, a whole-day nutrient broth medium bacterial growth was separated by centrifugation (at 4000 rpm for 10 min); cells were then cleaned with distilled water, fixed in 3% glutaraldehyde, rinsed in phosphate buffer, and post-fixed in potassium permanganate solution for 5 min. at room temperature. The samples were dehydrated for 15 min in each ethanol dilution, ranging from 10 to 90%, and then for 30 min in absolute ethanol. Samples were finally immersed in pure resin and gathered on thin copper grids. Then, sections were double stained in lead citrate and uranyl acetate. A JEOL - JEM 1010 transmission electron microscope at 70 kV was used to observe the stained sections at The Regional Center for Mycology and Biotechnology (RCMB), Al- Azhar University [33–[34]–35].

### Statistical analyses

Three repetitions were used for each experiment. Using the descriptive statistics frequencies and the Statistical Package for the Social Sciences (SPSS) version 25 statistical tool, the results were presented as the mean ± SD.

## Results and discussion

### Identification of bioactive compounds in the ***Ulva lactuca*** extract

#### GC analysis of ***U. lactuca*** methanolic extract

**Fig. 1:**
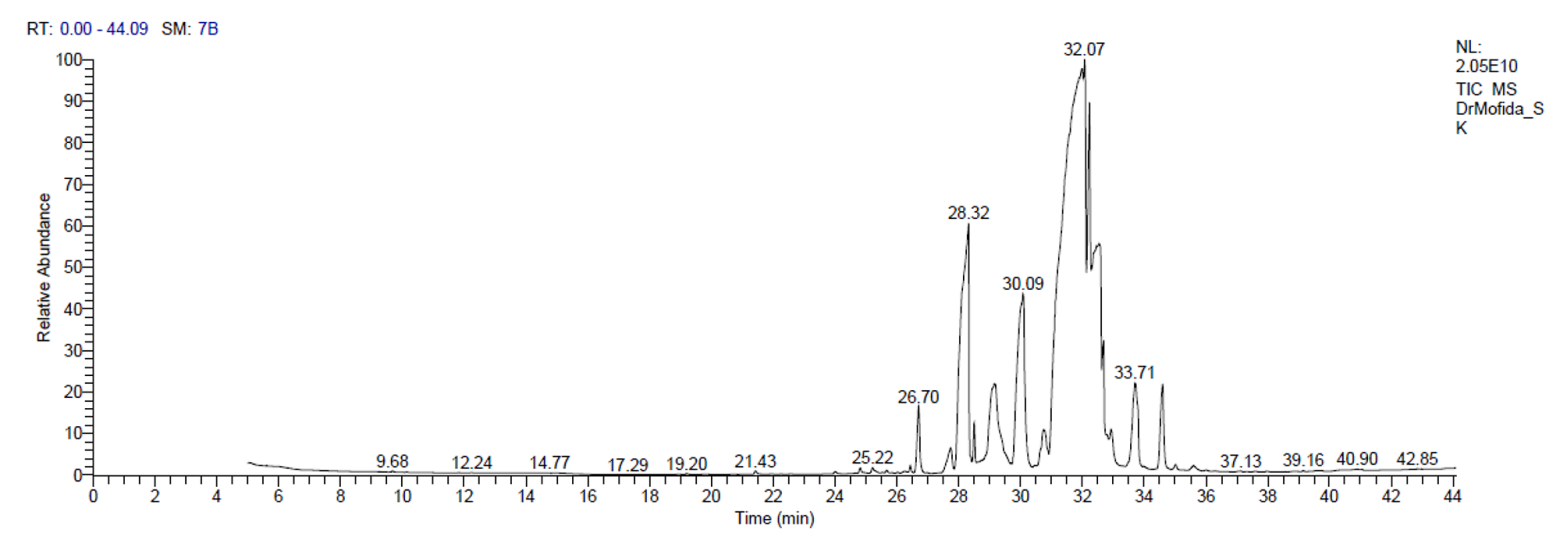
GC graph of *Ulva lactuca* methanolic extract

*Ulva lactuca* methanolic extract GC–MS analysis (Fig. 1; Table 1) revealed many components, which indicated the presence of several fatty acids, e.g., Oleic acid, Undecanoic acid, Ethyl linoleate, and 9-Dodecenoic acid that seem to be responsible for antibacterial activity as this result agrees with Amel et al. [36] who detected the antibacterial activity of *Ulva rigida* isolated fatty acid and found that the activity found in the seaweed samples seems to be caused by oleic, palmitic and stearic acids. The analysis also detects the presence of Hydrocarbons that are used as antibacterial, antimicrobial, and Terpenese as a good antimicrobial. Esters are used in medicine [37], and other components like alkaloids, polysaccharides, alcohols, and Phenolics. *U. lactuca* GC-MS detects the presence of Cefazolin, a first-generation cephalosporin and beta-lactam antibiotic with bactericidal action [38].

**Table 1:** Gas chromatography (GC) analysis of *Ulva lactuca* methanolic extract

RT	compound	Common name	Molecular formula	Peak area%	M.WT	Chemical group
24.81	3,10-Dioxatricyclo[4.3.1.0(2,4)]dec -7-ene	2-Hydroxyphenethyl alcohol	C_8_H_10_O_2_	0.12	138	phenol
31.14	Tricyclo[5.3.0.0(3,9)]decan-4-one	Thymol	C_10_H_14_O	2.85	150	
24.81	E-7-Tetradecenol	Tetradecanal	C_14_H_28_O	0.12	212	Fatty acid
26.70	10-Undecenoic acid, methyl ester	9-Dodecenoic acid	C_12_H_22_O_2_	1.91	198	
27.74	2-Nonanone, o-methylixime	Decanamide	C_10_H_21_NO	0.96	171	
27.74	2-Undecanon, o-methyloxim, (synoder anti)	Dodecanamide	C_12_H_25_NO	0.96	199	
30.74	Ethyl (9Z,12Z)-9,12-octadecadienoate #	Ethyl linoleate	C_20_H_36_O_2_	1.27	308	
30.74	8,11,14-Eicosatrienoic acid, (Z,Z,Z)-	Ethyl linolenate	C_20_H_34_O_2_	1.27	306	
28.32	4-Heptanol, 2,4-dimethyl-	Nonan-1-ol	C_9_H_20_O	21.15	144	
28.32	Undecanoic acid	Undecanoic acid	C_11_H_22_O_2_	21.15	186	
29.15	9-Octadecenoic acid (Z)-	Oleic acid	C_18_H_34_O2	1.87	282	
35.02	trans-2-undecenoic acid	2-Cyclopentylhexanoic acid	C_11_H_20_O_2_	0.15	184	
31.14	9,12,15-Octadecatrienoic acid, methyl ester, (Z,Z,Z)-	Methyl linolenate	C_19_H_32_O_2_	2.85	292	
32.07	9-Octadecenoic acid (Z)-, methyl ester	Methyl oleate	C_19_H_36_O_2_	5.22	296	
32.23	12-Tridecynoic acid, methyl ester	Megatomoic Acid	C_14_H_24_O_2_	4.01	224	
32.23	9,12-Octadecadienoic acid (Z,Z)-	Linoleic acid	C_18_H_32_O_2_	4.01	280	
31.14	9,12,15-Octadecatrienoic acid, methyl ester, (Z,Z,Z)-	Methyl linolenate	C_19_H_32_O_2_	2.85	292	
30.9	3,6-Octadecadienoic acid, methyl ester	Methyl linoleate	C_19_H_34_O_2_	11.82	294	
26.70	DL-Norleucine	1-Nitrohexane	C_6_H_13_NO_2_	1.91	131	Alkaloid
26.70	2,7-Dioxatricyclo[4.4.0.0(3,8)]deca n-4-amine, stereoisomer	Arecoline	C_8_H_13_NO2	1.91	155	
25.22	L-Valine, N-glycyl-	L-Theanine	C_7_H_14_N_2_O_3_	0.19	174	
25.22	4 H,5 H-Pyrano[4,3-d]-1,3-dioxin, tetrahydro-8a-methyl-	Isobutyl acetoacetate	C_8_H_14_O_3_	0.19	158	Carboxylic acid
29.08	4,6-O-Ethylidene-à-D-glucose	Diethyl tartrate	C_8_H_14_O_6_	2.27	206	
33.72	trans-Traumatic acid	Traumatic acid	C_12_H_20_O_4_	4.18	228	
26.44	Butane, 1,1-dibutoxy-	1,12-Dodecanediol	C_12_H_26_O_2_	0.14	202	Alcohol
30.9	9,12-Octadecadienal	Linolenyl alcohol	C_18_H_32_O	11.82	264	
28.32	2 H-thiopyran, 3-butyltetrahydro-	Cyclononane		21.15	158	Hydrocarbon
28.50	d-Glucosamine	D-Glucosamine	C_6_H_13_NO_5_	0.73	179	Amino acid
28.50	D-(-)-Norvaline	D-Norvaline	C_5_H_11_NO_2_	0.73	117	
28.50	D-Lysine	D-Lysine	C_6_H_14_N_2_O_2_	0.73	146	
29.15	2-Aminoethanethiol Hydrogen sulfate (ester)	1-Azaniumyl-2-sulfonatosulfanylethane	C_2_H_7_NO_3_S_2_	1.87	157	Ester
29.15	Alpha-l-rhamnopyranose	2-Deoxyhexopyranose	C_6_H_12_O_5_	1.87	164	Polysaccharide
32.69	Melezitose	Raffinose	C_18_H_32_O_16_	1.04	504	
32.00	(Z)6,(Z)9-Pentadecadien-1-ol	Dihydrofarnesol	C_15_H_28_O	30.22	224	Terpenes
31.14	E,E-6,11-Tridecadien-1-ol acetate	Curcumadiol	C_15_H_26_O_2_	2.85	238	
35.02	Cefazolin	Cefazolin	C_14_H_14_N_8_O_4_S_3_	0.15	454	Antibiotic

#### FTIR characterization of ***U. lactuca*** methanolic extract

**Fig. 2:**
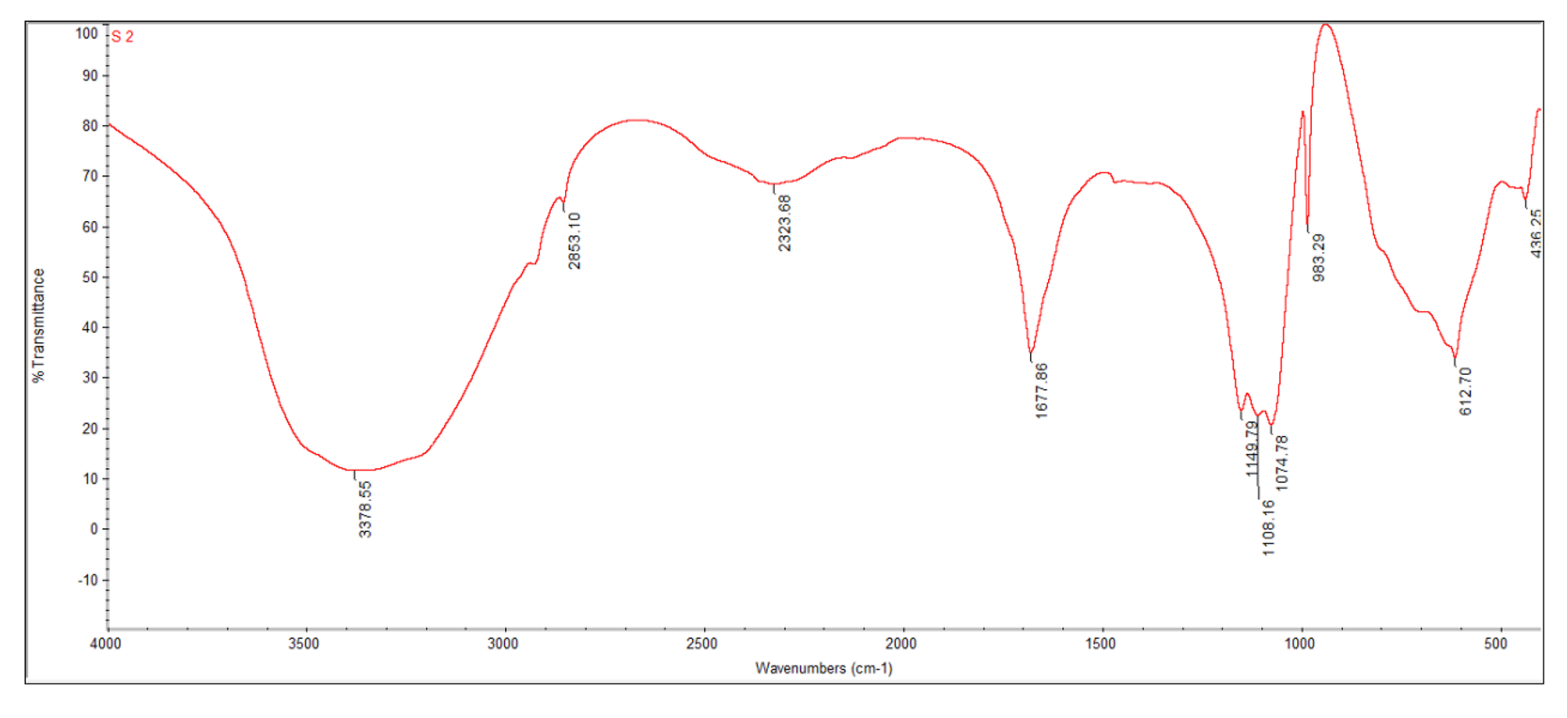
FTIR graph of *Ulva lactuca* methanolic extract

The functional group of the extract active components was determined using the FT-IR spectrum (Fig. 2), based on *U. lactuca* methanolic extract peak ratio. Analysis’s FT-IR findings revealed that the functional group at 612.70 is alkyl halides, the functional group at 993.29 is carboxylic acids, and the functional groups at 1074.78 and 1108.16 are aliphatic amines [39]. Carbohydrate ACH_2_OH groups are responsible for the absorption band at 1149.79 [40]. The bands at 1677.86 cm 1 of the spectra show C = O stretching of aromatic amide I (proteins and peptides) [41 − 39]. The functional group at 2323.68 is nitriles, the functional group at 2853.10 is alkanes, and the absorbance band at 3378.55 indicates that alcohols and phenols were present in the *U. lactuca* sample [39].

### Antibacterial activity

**Fig. 3:**
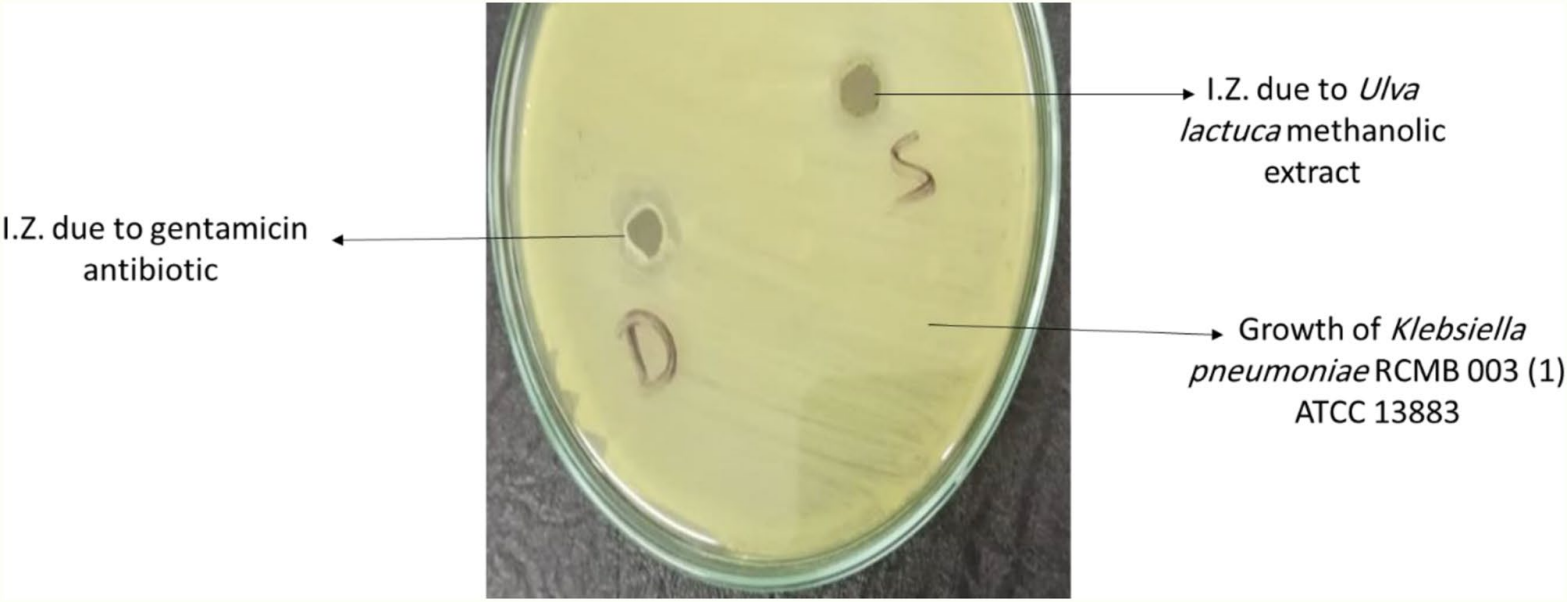
Antibacterial activity of *Ulva lactuca* methanolic extract (1.25 mg/ml) and gentamicin(4 µg/ml) against *Klebsiella pneumoniae* RCMB 003 (1) ATCC 13,883, D, is the inhibition zone (I.Z.) of the drug (gentamicin) and S, is the inhibition zone of the sample (*Ulva lactuca*).

Methanolic extract of the *Ulva lactuca* sample has a remarkable antibacterial action on *Klebsiella pneumoniae* RCMB 003 (1) ATCC 13,883, which was represented as 14.04 mm ± 0.376, On the other hand, the gentamicin antibiotic can control the growth of tested bacterial pathogen with stronger and larger I.Z. diameter of 20.91 mm ± 0.522366 (Fig. 3). Results were recorded in triplicate and represented as mean ± SD, as shown in Table 2. From our results, the methanolic extract of the *Ulva lactuca* seems to be a promising candidate for secure medical applications to prevent the growth of *Klebsiella pneumoniae* RCMB 003 (1) ATCC 13,883, as detected by the promising inhibition zone formed. This may stand for phenol and alcohol compounds (from FTIR analysis) of *Ulva* extract, which produced the antibacterial properties as mentioned in the data provided by Radhika and Amer [39] and stated that phenolic compounds exhibited good antimicrobial activities and noticed a good inhibition zone (5 mm ± 0.05) formed by *Ulva lactuca* against *Klebsiella pneumoniae*.

**Table 2:** Antibacterial activity of *Ulva lactuca* methanolic extract against *Klebsiella pneumoniae* RCMB 003 (1) ATCC 13,883

pathogenic bacterial strain	Inhibition zone (mm)	
	**Ulva lactuca methanolic sample**	**Gentamicin as a Control**
*Klebsiella pneumoniae* RCMB 003 (1) ATCC 13,883	14.04 ± 0.376652	20.91 ± 0.522366

Afifah et al. [42] deduced that Seaweed extracts demonstrated various bio-potential behaviors, including antibacterial ones against Gram-positive and Gram-negative bacteria.

### MIC determination

*U. lactuca* methanolic sample plus the antibiotic used (Gentamicin) MIC values against *Klebsiella pneumoniae* RCMB 003 (1) ATCC 13,883 were summarized in Table 3, in which 1.25 mg/ml of the algal extract could strongly inhibit the growth of *Klebsiella pneumoniae* RCMB 003 (1) ATCC 13,883, resulting in no visible growth. For that, two concentrations of 1.25 mg and 2.5 mg/ml of algal extract were suggested for this experiment in combination with antibiotics to achieve maximum inhibition of the bacterial pathogen (*Klebsiella pneumoniae* RCMB 003 (1) ATCC 13,883) in further experiments. Similarly, Chandrasekaran et al. [43] stated that (MIC) falls in the range of 125 µg/ml to 500 µg/ml. The ethyl acetate extract of *U. fasciata* showed the greatest mean of IZ (15.0 mm) and the lowest MIC (125 g/ml) against *B. subtilis*. Our results were accepted from the point of view of the study done by Choi et al. [44], as the 1/2 MIC of fucoidan completely inhibited the development of the tested bacteria, methicillin-resistant *Staphylococcus aureus*, whether it was given alone, in combination with oxacillin (1/2 MIC), or in combination with ampicillin (1/2 MIC).

**Table 3:** MIC of *Ulva lactuca* methanolic sample against *Klebsiella pneumoniae* RCMB 003 (1) ATCC 13,883

pathogenic bacterial strain	MIC of *Ulva lactuca* methanolic sample (mg/ml)	Gentamicin (4 µg/ml) as a Control
*Klebsiella pneumoniae* RCMB 003 (1) ATCC 13,883	1.25	21

**Table 4:** Diameter of inhibition zone (mm) with *Ulva lactuca* methanolic extract (2.5 mg/ml) and antibiotics individually and in combination

Pathogen	Diameter of Inhibition zone at Ulva lactuca methanolic extract concentration (2.5 mg/ml)						
*Klebsiella pneumoniae* RCMB 003 (1) ATCC 13,883	*Ulva*	Chloramphenicol (500 µg/ml)	*Ulva *+ Chloramphenicol (500 µg/ml)	Fold area increasing%	Gentamicin (4 µg/ml)	*Ulva* + Gentamicin (4 µg/ml)	Fold area increasing%
	14	14	18	28.571%	19	25	31.578%

**Table 5:** Diameter of inhibition zone (mm) with *Ulva lactuca* methanolic extract (1.25 mg/ml) and antibiotics individually and in combination

Pathogen	Diameter of Inhibition zone at Ulva lactuca methanolic extract concentration (1.25 mg/ml)						
*Klebsiella pneumoniae* RCMB 003 (1) ATCC 13,883	Ulva	Chloramphenicol (500 µg/ml)	*Ulva* + Chloramphenicol (500 µg/ml)	Fold area increasing%	Gentamicin (4 µg/ml)	*Ulva* + Gentamicin (4 µg/ml)	Fold area increasing%
	12	14	16	14.285%	19	22	15.789%

### Synergetic effect between ***Ulva lactuca*** extract and antibiotics against ***Klebsiella pneumoniae*** RCMB 003 (1) ATCC 13,883

**Fig. 4:**
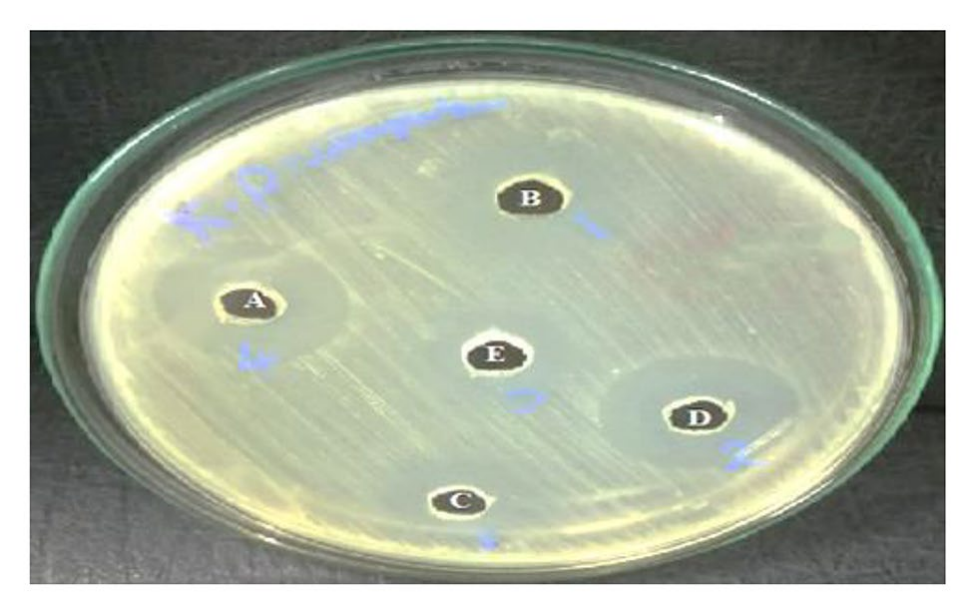
Synergetic inhibition effect of *Klebsiella pneumoniae* RCMB 003 (1) ATCC 13,883 by the action of *Ulva lactuca* methanolic extract with different antibiotics; A: algal extract (2.5 mg/ml) + gentamicin, B: algal extract (2.5 mg/ml) + chloramphenicol, C: algal extract (1.25 mg/ml) + chloramphenicol, D: algal extract (1.25 mg/ml) + gentamicin, E: algal extract only (2.5 mg/ml)

The combination of *Ulva lactuca* methanolic extract and an antibiotic can synergistically inhibit pathogenic bacteria *Klebsiella pneumoniae* RCMB 003 (1) ATCC 13,883. This was detected in our results. The mechanism underlying the synergistic activity is unknown. This study investigates the synergistic mechanism of two antibiotics (Chloramphenicol and Gentamicin) in combination with *Ulva* extract against the chosen pathogenic bacterium. There were three copies of each result. Around several combination wells, the inhibition zone’s average diameter in mm was measured. Our findings noted a promising synergetic effect appeared against *Klebsiella pneumoniae* RCMB 003 (1) ATCC 13,883, when mixing our algal extract with different antibiotics (Chloramphenicol and Gentamicin), as in Fig. (4). This was denoted by the highest increase in fold area at algal extract concentration (2.5 mg/ml) with gentamicin, which gives 31.578%, followed by adding chloramphenicol to algal extract, which records 28.571%, as shown in Table (4). But when decreasing the concentration of *Ulva lactuca* methanolic extract to (1.25 mg/ml) and mixing it with gentamicin, the fold area was 15.789%; however, it then decreased to 14.285% by adding chloramphenicol to the algal extract, Table (5).

Thus, our findings showed that antibiotics and *Ulva lactuca* methanolic extract combination could increase the antibiotic efficacy against the resistant pathogen (*Klebsiella pneumoniae* RCMB 003 (1) ATCC 13,883). The most likely reason for medicines’ improved antibacterial action when combined with Ulva *lactucae* methanolic extract may be attributed to what was mentioned by Choi et al. [44], that the outer membrane of Gram-ve bacteria serves as a defense against a variety of environmental elements, including antibiotics. The inhibitory effect of these antibiotics may be impacted by combinations of some herbal substances and certain antibiotics.

The *Ulva* extract and antibiotic combination may attach to the cell membrane, resulting in its lysis, resulting in cell entry, which causes the DNA to unwind, ultimately leading to cell death. On the other hand, this may also be due to the bonding reaction between the components of the algal extract and the antibiotics. Because of the potential for membrane disruption and cytoplasm leakage caused by this combination, antibiotics, and algae extract may be able to enter bacterial cells and damage DNA, the same mechanism was suggested by EL-Deeb et al. [32] about the bonding reaction between selenium- nanoparticles, and antibiotics. This was in accordance with the new findings done by Abdul-Rahim et al., [45], which mentioned that; metabolic responses to polymyxin B and/or chloramphenicol against (New Delhi- Metallo) NDM- producing *K. pneumoniae* is due to the inhibition of bacterial cell membrane formation, it also had a bad effect on arginine and nucleotides metabolism, and also affect glycolysis and pentose phosphate pathways.

### Transmission electron microscope

**Fig. 5:**
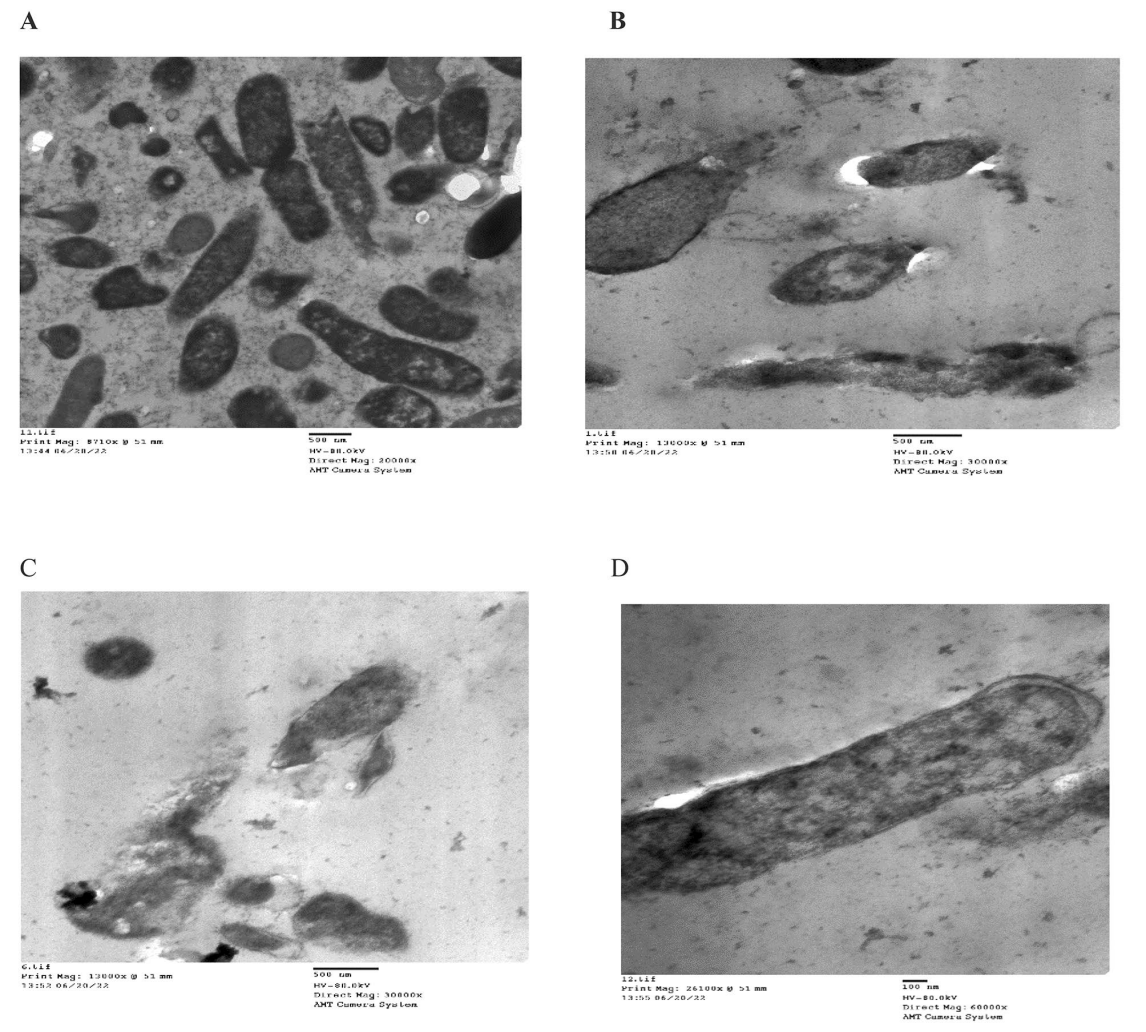
TEM micrographs of *Klebsiella pneumoniaee* RCMB 003 (1) ATCC 13,883 cells after treatment with methanol extract of *U. lactuca* at 2.5 mg/ml in- combination with gentamicin (4 µg/ml). **(A)** Untreated Cells, **(B);** Disintegration of the membrane layers, **(C);** Unknown small particles outside the cell wall, broken cells and cytoplasmic leakage resulted in a significant reduction in the cell’s size, **(D);** Elongated cell with thickened cell wall

The TEM data of *Klebsiella pneumoniae* RCMB 003 (1) ATCC 13,883 treated *U. lactuca* methanolic extract provide a clearer picture and a deeper knowledge of cellular morphological degenerations. Figure 5 (A, B, and C). *Klebsiella pneumoniae* RCMB 003 (1) ATCC 13,883 cells in the control sample were visible in micrographs (Fig. 5A). These cells displayed the characteristic round to elliptical forms and were encircled by the inner and outer layers. [46]. Although some of the individual cells were in cell division middle of the constriction process, all of them appeared to be healthy and free of cellular damage (Fig. 5A). But exposing the bacterial cells to the *U. lactuca* methanol extract (2.5 mg/mL) in combination with gentamicin (4 µg/mL) has caused bacterial envelope layers lysis, (Fig. 5B). Also, we notice the appearance of unknown small particles that adsorbed on the outer membrane, and there are some cells occur with wavy shaped envelop layers (comparing to control cell) and the presence of unknown small particles Fig. 5B C, respectively. Figure 5 C showed the disruption of some cell walls, which in turn lead to leakage of cell cytoplasm. Figure 5D showed an Elongated cell with a thickened cell wall, this thing has been confirmed by Supardy et al. [47], who stated that *Halimeda discoidea* hexane extract-treated *K. pneumoniae* ATCC 13,883 cells had strange morphologies of enlargement, shape irregularity, size decrement, and other various abnormalities that weren’t present in untreated control cells. Treatments that result in the cells contracting and changing shape demonstrated the stressed conditions in which the cells were placed. Rajeshwari et al. [48] reported that the *K. pneumoniae* cell wall disintegration effect of antibiotics such as cefotaxime was due to the active bind of such antibiotics with penicillin-binding proteins and that caused bacterial cell wall cross-linking peptidoglycan units disorganization causing weekend for such linkage, so any treatment occur after such binding weekend will in turn break bacterial membrane easily causing cells’ perforation and the cytoplasm leakage. Supardy et al. [47] illustrated that the cell morphology deviation from the normal short-rod shape may be due to the changes in cell enzymes or proteins.

Spratt [49] and Satta et al. [50] found that penicillin and mecillinam antibiotic action occurs through their binding to Gram-negative cellular enlargement and division enzymes and proteins, which in turn caused cell lysis and death. Derakhshan et al. [51] also recorded *K. pneumoniae* ATCC 13,883 cells elongation after the treatment with cumin herb (*Cuminum cyminum* L.) extract. Also, the presence of Cefazolin, identified by GC-MS analysis, may be the cause of the tested extract’s antibacterial action. Penicillin-binding proteins (PBP) on the bacterial cell wall’s inner membrane are bound by cefazolin and rendered inactive. PBP inactivation hinders the cross-linking of peptidoglycan chains, which is essential for the strength and stiffness of bacterial cell walls. This has a role as an antibacterial medicine because it weakens the bacterial cell wall and induces cell lysis [38].

## Conclusion

*Ulva lactuca* methanolic extract " for the first time” exerted promising antibacterial activity and synergistic effects when administered with gentamicin or chloramphenicol “that are from the most popular antibiotics used in the Egyptian society” against *Klebsiella pneumoniae* RCMB 003 (1) ATCC 13,883 be helpful for usage as a natural product agent to combat infections that are resistant to antibiotics. We recommend that further study should be done on this promising algal extract, with the evaluation of its cytotoxicity against any normal cell line.

## Data Availability

The datasets used and/or analyzed during the current study available from the corresponding author on reasonable request.
